# The association between kidney function-standardized serum uric acid levels and stroke risk: insights from the National Health and Nutrition Examination Survey

**DOI:** 10.3389/fnagi.2025.1542298

**Published:** 2025-04-29

**Authors:** Xinmin Deng, Kunlin Kuang, Yumei Zhong, Rui Lai, Xiaofeng Lv, Shanshan Liu, Meijun Liu, Jingtao Liang, Dongdong Yang

**Affiliations:** ^1^School of Clinical Medicine, Chengdu University of Traditional Chinese Medicine, Chengdu, China; ^2^School of Acupuncture and Tuina, Chengdu University of Traditional Chinese Medicine, Chengdu, China; ^3^Department of Pain, Chengdu Integrated Traditional Chinese Medicine and Western Medicine Hospital, Chengdu, China; ^4^Department of Neurology, Hospital of Chengdu University of Traditional Chinese Medicine, Chengdu, China

**Keywords:** serum uric acid, serum creatinine, stroke, NHANES, cross-sectional study

## Abstract

**Background:**

Previous studies on the relationship between serum uric acid to serum creatinine ratio (SUA/SCr) and stroke have shown inconsistent results. This study investigates the association between SUA/SCr and stroke risk using data from the National Health and Nutrition Examination Survey (NHANES).

**Materials and methods:**

A cross-sectional analysis was conducted using NHANES data from 1999 to 2018. Weighted univariate and multivariate logistic regression models were used to evaluate the association between SUA/SCr and stroke. The restricted cubic spline (RCS) curve was used to explore the nonlinear relationship between SUA/SCr and stroke risk.

**Results:**

In the regression model adjusted for all covariates, the OR (95% CI) for the association between SUA/SCr and stroke was 0.84 (0.78, 0.92), indicating a significant association between SUA/SCr and stroke risk. When SUA/SCr was analyzed as a categorical variable by quartiles, participants in the highest SUA/SCr quartile (Q4) had a 53% lower risk of stroke compared to those in the lowest quartile (Q1), with an OR (95% CI) of 0.47 (0.31, 0.71). RCS analysis revealed a nonlinear relationship between SUA/SCr and stroke risk (nonlinearity *p* = 0.048). Before the nonlinear inflection point (6.33), stroke risk significantly decreased as SUA/SCr increased. After this point, the decrease in stroke risk with increasing SUA/SCr slowed down markedly.

**Conclusion:**

Our study indicates that higher SUA/SCr levels are associated with a lower risk of stroke. However, further prospective longitudinal studies are required to establish the causal relationship and explore the potential role of SUA/SCr in stroke risk assessment and prevention strategies.

## 1 Introduction

Stroke is a major global health concern, ranking as the second leading cause of death and the third leading cause of disability worldwide. It typically manifests as a sudden neurological deficit due to an interruption in cerebral blood flow, often resulting in long-term disability, reduced quality of life, and a significant medical burden (Benjamin et al., 2017; GBD 2016 Stroke Collaborators, 2019; Campbell and Khatri, 2020; Zhang et al., 2023; Martin et al., 2024). US National Health and Nutrition Examination Survey (NHANES) data from 2017 to 2020 show that approximately 9.4% of US adults aged 20 and older reported a history of stroke (Martin et al., 2024). Projections indicate that by 2030, the number of US adults aged 18 and older will increase by 3.4 million, accounting for 3.9% of the adult population, representing a 20.5% increase in prevalence compared to 2012 (Martin et al., 2024). This underscores the urgent need for effective prevention and treatment strategies.

Among the many potential risk factors for stroke, hyperuricemia has garnered increasing attention. Serum uric acid (SUA), the end product of purine metabolism, has been linked to cardiovascular diseases such as hypertension, atherosclerosis, and ischemic heart disease, all of which are known risk factors for stroke (Waring et al., 2000; Chang et al., 2018; Kimura et al., 2021; Saito et al., 2021). While uric acid has traditionally been viewed as a harmful pro-oxidant, it also exhibits antioxidant properties under certain conditions, suggesting a more complex role in the pathophysiology of vascular diseases (Sautin and Johnson, 2008; Neogi et al., 2012; Kang and Ha, 2014). However, endogenous SUA levels are largely influenced by renal clearance capacity; therefore, it is essential to consider kidney function when evaluating the relationship between SUA and various diseases, particularly cardiovascular diseases (Li F. et al., 2018). The serum uric acid to serum creatinine ratio (SUA/SCr) has emerged as a novel biomarker that may better reflect the dynamic interplay between uric acid metabolism and renal function. Creatinine, a byproduct of muscle metabolism, is commonly used as a marker of kidney function. The SUA/SCr ratio adjusts for renal clearance, providing a more comprehensive assessment of uric acid’s role in vascular health (Toyama et al., 2015; Chen et al., 2022; Kawamoto et al., 2023). In recent years, numerous studies have examined the relationship between SUA/SCr and various cardiovascular diseases, including stroke, but the results have been inconsistent. Some studies suggest that higher SUA/SCr levels are associated with an increased risk of stroke and other cardiovascular diseases (Wang et al., 2021; Sun et al., 2022), as well as adverse outcomes, while other studies have reported a protective effect (Liu et al., 2023; Tang et al., 2024; Zhang et al., 2024). These discrepancies may be attributed to differences in population characteristics (e.g., age range, ethnic composition), regional variations, and methodological heterogeneity, including discrepancies in stroke definitions, sample sizes, and statistical approaches.

Given the growing interest in the relationship between SUA/SCr and cardiovascular outcomes, including stroke, this study aims to explore the association between SUA/SCr and stroke in a nationally representative sample from the NHANES. By leveraging this large, publicly available dataset, we aim to provide further insights into the potential utility of the SUA/SCr ratio as a predictive marker for stroke risk.

## 2 Materials and methods

### 2.1 Study population

NHANES is a national health survey conducted by the Centers for Disease Control and Prevention (CDC) and the National Center for Health Statistics (NCHS), aimed at collecting data from a nationally representative sample, including demographics and individual nutritional status, and more.^1^ Our analysis included individuals from the 1999 to 2018 NHANES cycles. The study protocol was approved by the NCHS Research Ethics Review Board, and all participants provided written informed consent.

**FIGURE 1:**
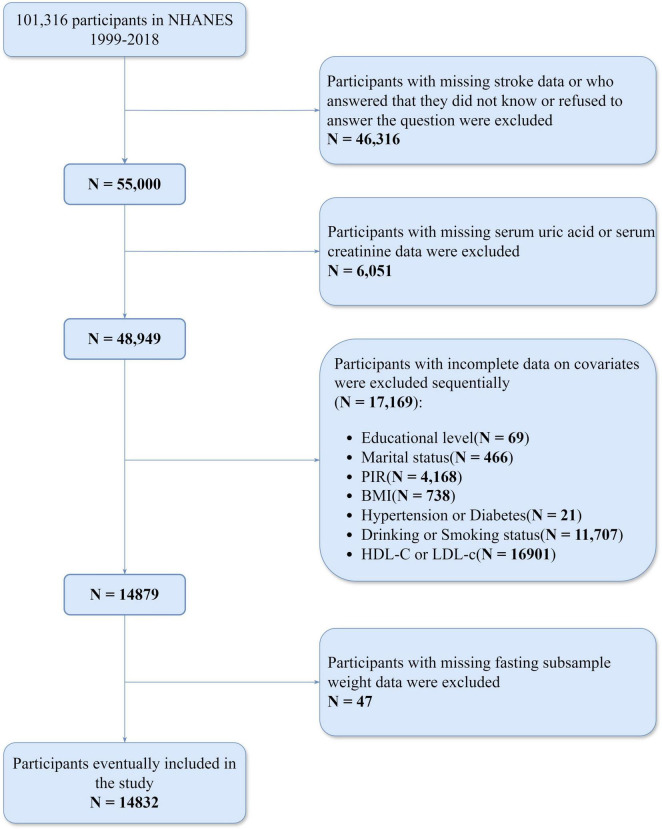
Participant selection flowchart.

Initially, the study identified 101,316 participants from the NHANES dataset (1999–2018). We sequentially excluded 46,316 participants who either had missing responses to stroke-related questions, answered “Don’t know,” or refused to answer; 6,051 participants with missing serum uric acid or serum creatinine data; 69 participants with missing education level data; 466 participants missing marital status data; 4,168 participants lacking PIR data; 738 participants missing BMI data; 21 participants missing hypertension or diabetes data; and 11,707 participants missing alcohol or smoking data; 16,901 participants with missing HDL-c or LDL-c data. Then, we further excluded 47 participants with missing fasting subsample weight data. As a result, a total of 14,832 participants were included in the study (Figure 1).

### 2.2 Assessment of variables

In this study, stroke was considered the outcome variable. Stroke was assessed through the NHANES self-reported questionnaire, which asked: “Has a doctor or other health professional ever told you that you had a stroke?” Participants could choose from four responses: “Yes,” “No,” “Refused,” or “Don’t know.” Those who answered “Yes” were classified as stroke patients, while those who answered “No” were classified as non-stroke patients.

SUA/SCr as the independent variable was calculated as SUA divided by SCr. Serum uric acid was measured using an enzymatic method (Beckman UniCel^®^ DxC 800, Beckman Coulter Inc.) in NHANES 2007–2018, while earlier cycles employed a similar enzymatic or uricase-based colorimetric method. Serum creatinine was determined by a modified kinetic rate Jaffe method in NHANES 1999–2006 and an enzymatic assay in subsequent cycles. Detailed protocols can be found on the NHANES website. All analyses were calibrated for method and instrument changes to ensure comparability across survey years.

We selected covariates based on both clinical relevance and data availability across multiple NHANES cycles. The covariates in this study include age, race, education level, body mass index (BMI), poverty-income ratio (PIR), marital status, smoking and drinking habits, diabetes, hypertension, high density lipoprotein cholesterol (HDL-c), and low density lipoprotein cholesterol (LDL-c), all of which may act as potential confounding factors influencing the relationship between SUA/SCr and stroke. In addition, lifestyle factors such as diet and physical exercise are also associated with stroke risk. However, these variables were not consistently or comprehensively recorded across all NHANES cycles and often had substantial missing data. Considering that including these variables would significantly reduce the sample size of stroke patients and potentially introduce additional bias, we did not include them as covariates in this study. Race was divided into Mexican American, non-Hispanic White, and non-Hispanic Black groups. Age was categorized as either < 60 or ≥ 60 years. Participants who were married or living with a partner were classified as “with partner,” while those who were widowed, divorced, separated, or never married were categorized as “without partner.” Education was divided into three levels: less than high school, high school graduate, and more than high school. BMI was categorized as < 25, 25–29.99, and ≥ 30 kg/m^2^. PIR was categorized as < 1.30, 1.31–3.49, or ≥ 3.50, corresponding to low, middle, and high income, respectively. Participants who had smoked at least 100 cigarettes in their lifetime were classified as smokers. Participants who had at least 12 drinks in any given year or had ever had 4/5 drinks or more per day were categorized as drinkers. An average systolic blood pressure (SBP) of at least 140 mmHg and/or an average diastolic blood pressure (DBP) of at least 90 mmHg, as well as self-reported diagnosis of hypertension and antihypertensive drug use, were considered hypertension (Reboussin et al., 2018; Flack and Adekola, 2020). Participants were defined as diabetic if they had been diagnosed by a physician, had a hemoglobin A1c level above 6.5%, fasting blood glucose level ≥ 7.0 mmol/L, random blood glucose level ≥ 11.1 mmol/L, or were using diabetes medications or insulin (American Diabetes Association Professional Practice Committee, 2022).

### 2.3 Statistical analyses

To account for the complex, multistage sampling design of NHANES, we applied fasting subsample weights as recommended by the NHANES guidelines and conducted weighted analyses to enhance data accuracy. Continuous variables are presented as medians with interquartile ranges, while categorical variables are shown as counts with corresponding percentages. Baseline characteristics of participants were then compared by stroke status using the Kruskal–Wallis and chi-square tests. To estimate the adjusted odds ratios (ORs) and their 95% confidence intervals (CIs) for SUA/SCr quartiles, weighted logistic regression models were employed. Three models were constructed: Model 1 had no adjustments; Model 2 was adjusted for age, race, marital status, education level, and PIR; and Model 3 included further adjustments for BMI, hypertension, diabetes, smoking status, alcohol consumption, HDL-c, and LDL-c. Weighted restricted cubic splines (RCS) were also used to clarify the dose-response relationship between SUA/SCr and stroke risk, adjusting for potential confounders. To explore potential differential associations within subgroups, we stratified participants by age, gender, race, BMI, smoking status, alcohol intake, hypertension, and diabetes, followed by interaction analyses. Statistical analyses were conducted using R software (version 4.4.1), with statistical significance set at a two-sided *P*-value of less than 0.05. The “survey” package (version 4.4-2) was employed for the complex sampling design, and the “rms” package (version 6.8-2) was used to fit RCS models.

## 3 Results

### 3.1 Baseline characteristics of participants

A total of 14,832 eligible participants, aged 20 to 85 years, were included in the final analysis. As shown in Table 1, among these participants, 516 self-reported having had a stroke. On average, participants with a history of stroke were older. The median SUA/SCr for participants with a stroke history was 5.98, while for those without a history of stroke, the median was 6.33. In the stroke group, approximately 8.68% had an education level below high school, 29.94% had a household income classified as low, and 43.24% had a BMI ≥ 30 kg/m^2^. Additionally, 71.90% of the participants in the stroke group had a history of smoking, while 82.03% had a history of alcohol consumption. Among participants with a history of stroke, 25.45% were diagnosed with diabetes, and 74.98% had hypertension. Furthermore, their median LDL-c and HDL-c levels were 2.79 mmol/L and 1.29 mmol/L, respectively, whereas in participants without stroke, the median LDL-c and HDL-c levels were 2.95 mmol/L and 1.32 mmol/L, respectively. Significant statistical differences were found between the two groups in terms of age, race, education level, PIR, BMI, LDL-c, smoking status, alcohol consumption, and the prevalence of hypertension, diabetes, and SUA/SCr levels (*P* < 0.05).

**TABLE 1:** Baseline characteristics of participants.

Variable	Total 14,832 (100%)	Non-stroke 14,316 (96.52%)	Stroke 516 (3.48%)	*P*
Age	46 (33, 59)	45 (33, 58)	65 (53, 74)	<0.001
Gender				0.200
Male	7,852 (51.30%)	7,566 (51.41%)	286 (47.18%)	
Female	6,980 (48.70%)	6,750 (48.59%)	230 (52.82%)	
Race				0.012
Mexican American	2,487 (7.27%)	2,433 (7.36%)	54 (4.10%)	
Non-Hispanic White	7,446 (72.58%)	7,169 (72.60%)	277 (71.55%)	
Non-Hispanic Black	2,786 (10.00%)	2,652 (9.89%)	134 (14.19%)	
Other race	2,113 (10.16%)	2,062 (10.15%)	51 (10.17%)	
Marital status				0.700
With partner	5,620 (34.30%)	5,409 (34.32%)	211 (33.44%)	
Without partner	9,212 (65.70%)	8,907 (65.68%)	305 (66.56%)	
Education level				<0.001
< High school	1,469 (4.84%)	1,389 (4.74%)	80 (8.68%)	
Completed high school	2,193 (11.14%)	2,076 (10.92%)	117 (19.39%)	
> High school	11,170 (84.02%)	10,851 (84.34%)	319 (71.92%)	
PIR				<0.001
Low income	4,203 (19.20%)	4,007 (18.92%)	196 (29.94%)	
middle income	5,645 (36.13%)	5,426 (35.95%)	219 (42.69%)	
high income	4,984 (44.67%)	4,883 (45.13%)	101 (27.37%)	
BMI				0.007
< 25	4,490 (31.89%)	4,359 (32.04%)	131 (26.40%)	
25–29.99	5,095 (33.99%)	4,932 (34.09%)	163 (30.36%)	
≥ 30	5,247 (34.12%)	5,025 (33.87%)	222 (43.24%)	
Smoke status				<0.001
No	7,031 (48.16%)	6,878 (48.69%)	153 (28.10%)	
Yes	7,801 (51.84%)	7,438 (51.31%)	363 (71.90%)	
Drinking status				0.014
No	2,381 (13.49%)	2,284 (13.37%)	97 (17.97%)	
Yes	12,451 (86.51%)	12,032 (86.63%)	419 (82.03%)	
Diabetes				<0.001
No	13,206 (92.00%)	12,840 (92.46%)	366 (74.55%)	
Yes	1,626 (8.00%)	1,476 (7.54%)	150 (25.45%)	
Hypertension				<0.001
No	8,553 (62.68%)	8,442 (63.69%)	111 (25.02%)	
Yes	6,279 (37.32%)	5,874 (36.31%)	405 (74.98%)	
LDL-c	2.95 (2.38, 3.57)	2.95 (2.38, 3.57)	2.79 (2.17, 3.47)	0.007
HDL-c	1.32 (1.10, 1.63)	1.32 (1.11, 1.63)	1.29 (1.06, 1.63)	0.200
SUA/SCr (continuous)	6.32 (5.35, 7.45)	6.33 (5.36, 7.46)	5.98 (4.83, 7.00)	<0.001
SUA/SCr (categorical)				<0.001
Q1	3,800 (24.98%)	3,609 (24.72%)	191 (34.84%)	
Q2	3,581 (25.02%)	3,455 (25.05%)	126 (23.81%)	
Q3	3,583 (25.00%)	3,474 (25.02%)	109 (23.98%)	
Q4	3,868 (25.01%)	3,778 (25.21%)	90 (17.37%)	

### 3.2 Association of SUA/SCr with stroke

We employed weighted univariate and multivariable logistic regression analysis to examine the relationship between SUA/SCr levels and stroke risk across various models. As shown in Table 2, both univariate and multivariate weighted logistic regression models indicated a negative association between SUA/SCr levels and stroke risk. To enhance the robustness of the analysis, SUA/SCr was also categorized into quartiles. In Model 3, after adjusting for all potential covariates, participants in the highest quartile of SUA/SCr (Q4) had a 53% lower risk of stroke compared to those in the lowest quartile (Q1) (Q4 vs. Q1, OR: 0.47; 95% CI: 0.31–0.71; *P* < 0.001).

**TABLE 2:** The association between SUA/SCr and stroke.

Characteristics	Model 1		Model 2		Model 3	
	OR (95% CI)	*P*	OR (95% CI)	*P*	OR (95% CI)	*P*
Continuous	0.83 (0.75, 0.91)	<0.001	0.88 (0.80, 0.96)	0.0037	0.84 (0.78, 0.92)	<0.001
**Quartile**						
Q1	Ref	Ref	Ref	Ref	Ref	Ref
Q2	0.67 (0.49, 0.93)	0.0163	0.80 (0.57, 1.12)	0.1846	0.79 (0.57, 1.11)	0.1753
Q3	0.68 (0.48, 0.96)	0.0271	0.82 (0.58, 1.16)	0.2623	0.74 (0.53, 1.04)	0.0833
Q4	0.49 (0.33, 0.72)	<0.001	0.58 (0.39, 0.86)	0.0076	0.47 (0.31, 0.71)	<0.001
*P* for trend		<0.001		0.0082		<0.001

**FIGURE 2:**
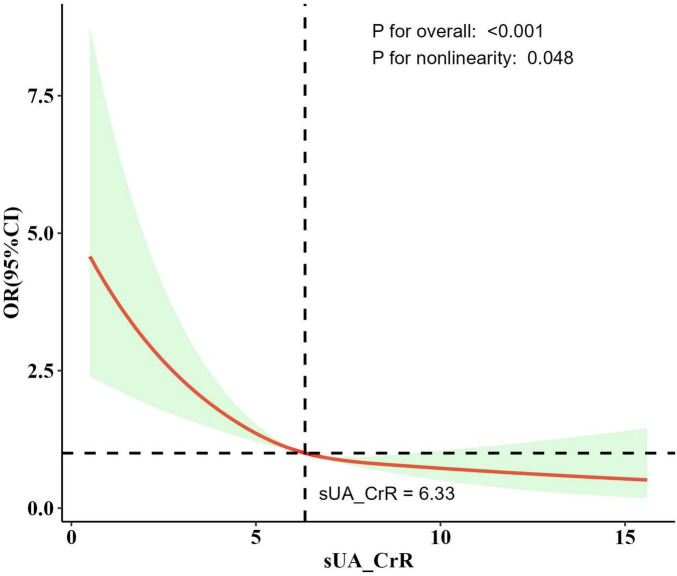
The dose-response relationship between SUA/SCr and stroke risk derived from the RCS model. Model adjusted for age, race, marital status, education level, poverty-income ratio, BMI, smoking status, alcohol consumption, hypertension, diabetes, HDL-c, and LDL-c. The solid line represents the adjusted odds ratio, and the shaded region indicates the 95% confidence interval. An inflection point around SUA/SCr = 6.33 was observed, after which the decline in stroke risk slowed. *P* for overall indicates the overall significance of the mode. *P* for nonlinearity indicates the significance of the nonlinear relationship of the model.

Dose-response curve analysis using RCS revealed a nonlinear relationship between SUA/SCr levels and stroke risk (*P* for overall < 0.001; *P* for nonlinearity = 0.048; Figure 2). Prior to the inflection point of this nonlinear relationship (6.33), an increase in SUA/SCr was associated with a significant decrease in stroke risk. Beyond the inflection point, the rate of decline in stroke risk reduction diminished as SUA/SCr continued to rise.

### 3.3 Subgroup analysis

**FIGURE 3:**
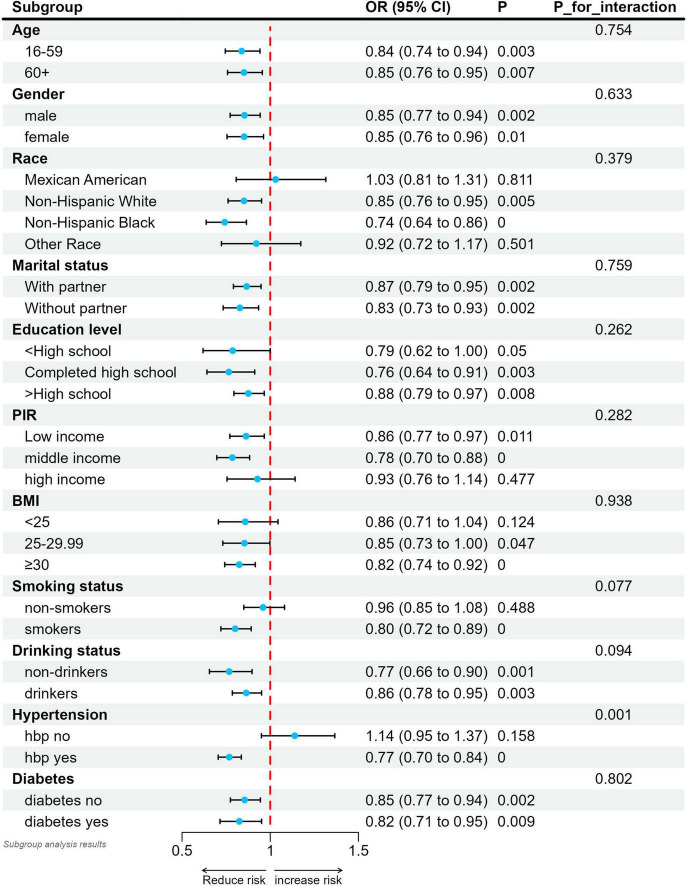
Relationship between SUA/SCr and stroke in each subgroup. Each subgroup was adjusted for all factors except the grouping factor itself. Solid circles indicate point estimates and horizontal lines indicate 95% confidence intervals. *P* for interaction were used to assess whether the correlations between the different subgroups were significantly different.

We performed a stratified analysis to assess whether the relationship between SUA/SCr and stroke differs across various subgroups (Figure 3). Our findings suggest an interaction between hypertension and SUA/SCr (interaction *P* < 0.05). Specifically, after adjusting for covariates, the negative association between SUA/SCr and stroke was more pronounced in hypertensive patients compared with nonhypertensive patients. In other subgroup analyses, no significant differences were observed in the relationship between SUA/SCr and stroke risk (interaction *P* > 0.05). Moreover, except for a positive but non-significant correlation between SUA/SCr and stroke in individuals without hypertension, all other subgroup analyses demonstrated a negative association between SUA/SCr and stroke risk.

## 4 Discussion

In this study, we investigated the association between the SUA/SCr and the risk of stroke using data from the NHANES public database. Our findings indicate that an increase in the SUA/SCr ratio is associated with a decreased risk of stroke.

Previous studies have explored the relationship between SUA and both stroke risk and outcomes, but the results have been inconsistent. Many scholars believe that these inconsistent findings may be due to the fact that SUA excretion by the kidneys typically depends on renal function, and patients with impaired renal function are at higher risk for elevated uric acid levels. Impaired renal function may be one of the key confounding factors in studies examining the association between SUA and stroke risk and outcomes. In this context, the SUA/SCr, which normalizes SUA levels to renal function, has been proposed in numerous studies as a potentially more accurate indicator of endogenous SUA levels (Li M. et al., 2018; Zhong et al., 2022; Sookoian and Pirola, 2023).

However, recent studies exploring the relationship between SUA/SCr and stroke have still yielded conflicting results. A prospective cohort study conducted by Wang et al. (2021) followed 96,378 participants from a community in China for a median of 11.7 years. The study found that SUA/SCr levels were positively associated with the risk of various cardiovascular diseases, including stroke (Wang et al., 2021). A single-center study conducted by Sun et al. (2022) retrospectively analyzed 428 young patients with first-time ischemic stroke over a median follow-up period of 3.14 years. The study found that SUA/SCr was positively associated with the risk of stroke recurrence (Sun et al., 2022). The aforementioned two studies show the adverse effects of high SUA/SCr levels on stroke. In contrast, another multicenter study conducted by Zhang et al. (2024) in China, involving 2,294 stroke patients, found that an increase in SUA/SCr was significantly associated with a reduced risk of stroke recurrence. This association remained consistent across subgroup analyses, including different age groups (Zhang et al., 2024). Additionally, two other cohort studies observed that higher SUA/SCr levels were associated with better neurological outcomes after stroke (Gong et al., 2022; Liu et al., 2023). These studies suggest a protective effect of high SUA/SCr levels on stroke outcomes. Although the studies mentioned above were all conducted in the same country, differences in study design, analytical methods, and the geographical distribution of study populations may explain the inconsistent results. Our study found a nonlinear negative correlation between SUA/SCr levels and stroke risk, which to some extent supports the protective effect of higher SUA/SCr levels against stroke reported in previous research. Moreover, further subgroup analyses appear to provide a potential explanation for the conflicting findings in previous studies regarding SUA/SCr and stroke risk. For instance, the inverse association was more pronounced in participants with hypertension compared to those without, indicating that differences in comorbid conditions or baseline risk profiles may partially account for the conflicting results reported across studies. Future studies could benefit from stratifying participants by key factors (e.g., hypertension, diabetes) to clarify these population-specific mechanisms.

The potential mechanisms between SUA/SCr and stroke occurrence and progression are not yet fully understood. The observed inverse association between SUA/SCr and stroke risk may be partially explained by the antioxidant role of uric acid, particularly under conditions where kidney function allows for effective clearance of excess uric acid. It is well known that endothelial dysfunction is a key factor in the pathogenesis of stroke, and oxidative stress plays an important role in this process (Little et al., 2021; Xu et al., 2021; Gupta et al., 2022; Higashi, 2022). While uric acid can act as a pro-oxidant in certain contexts, it is also recognized as a potent antioxidant. Uric can scavenge various reactive oxygen species (ROS), including hydroxyl radicals and peroxynitrite, thereby mitigating oxidative stress (So and Thorens, 2010; Leira et al., 2023). This antioxidant property may inhibit the progression of oxidative stress-associated vascular injury and help protect endothelial integrity, thereby reducing the risk of stroke. Because the SUA/SCr ratio inherently accounts for renal function, a moderately elevated ratio in individuals with intact excretory capacity may reflect a balance in which uric acid’s antioxidant properties outweigh any pro-oxidant effects (Al-Daghri et al., 2017). Some studies have further suggested that exogenous uric acid administration might minimize blood–brain barrier disruption and tissue injury in experimental stroke models, echoing the observed protective trend in humans (Amaro et al., 2019; Aliena-Valero et al., 2021). However, more research is needed to validate these mechanisms.

The strength of this study lies in its use of data from the NHANES database, which provides a sufficiently large sample size and thus offers adequate statistical power to support the findings. However, this study also has several limitations. First, the cross-sectional design restricts our ability to establish causal relationships. Longitudinal studies are needed to further verify whether SUA/SCr levels and their dynamic changes can predict stroke risk. Second, our data rely on self-reported stroke histories, which may introduce recall bias or misclassification. Although NHANES employs standardized questionnaires, some individuals may inaccurately recall past medical diagnoses, leading to either underestimation or overestimation of stroke prevalence. This limitation underscores the need for prospective studies with validated clinical endpoints. Third, the NHANES dataset primarily includes data from the US population, which may limit the generalizability of our findings to other racial and ethnic groups. Fourth, since NHANES does not differentiate between ischemic and hemorrhagic stroke or provide information on other subtypes, we were unable to further explore the association between SUA/SCr and specific stroke subtypes. Future studies that incorporate detailed stroke subtype information would be beneficial for clarifying these relationships and provide more important clinical insights. Furthermore, as the NHANES sample is drawn from a nationwide population, and considering that several observational studies conducted in different regions of China have shown inconsistent results, it is necessary to investigate whether regional differences in the US affect the association between SUA/SCr and stroke. Lastly, although adjustments were made for potential confounding factors, residual confounding cannot be entirely ruled out.

## 5 Conclusion

In conclusion, the study indicates that higher SUA/SCr levels are associated with a lower risk of stroke. However, further prospective longitudinal studies are required to establish the causal relationship and explore the potential role of SUA/SCr in stroke risk assessment and prevention strategies.

## Data Availability

Publicly available datasets were analyzed in this study. This data can be found here: https://www.cdc.gov/nchs/nhanes/index.htm.

## Footnotes

1
www.cdc.gov/nchs/nhanes/
